# Vesicomyidae (Bivalvia): Current Taxonomy and Distribution

**DOI:** 10.1371/journal.pone.0009957

**Published:** 2010-04-01

**Authors:** Elena M. Krylova, Heiko Sahling

**Affiliations:** 1 Laboratory of Ocean Benthic Fauna, P.P. Shirshov Institute of Oceanology, Moscow, Russia; 2 Department of Geosciences, MARUM-Center for Marine Environmental Sciences, University of Bremen, Bremen, Germany; University of Canterbury, New Zealand

## Abstract

Vesicomyid bivalves are a consistent component of communities of sulphide-rich reducing environments distributed worldwide from 77° N to 70°S at depths from 100 to 9050 m. Up-to-now the taxonomy of the family has been uncertain. In this paper, the current state of vesicomyid taxonomy and distribution at the generic rank are considered. This survey is founded on a database including information both from literature sources and also unpublished data of the authors on all recent species of vesicomyids. We suggest that the Vesicomyidae is not a synonym of Kelliellidae, and is therefore a valid family name. We propose to divide the family Vesicomyidae into two subfamilies: Vesicomyinae and Pliocardiinae. The Vesicomyinae includes one genus, *Vesicomya*, which comprises small-sized bivalves characterized by non-reduced gut and the absence of subfilamental tissue in gills. Symbiosis with chemoautotrophic bacteria has, so far, not been proved for *Vesicomya* and the genus is not restricted to sulphide-rich reducing habitats. The subfamily Pliocardiinae currently contains about 15 genera with mostly medium or large body size, characterized by the presence of subfilamental tissue in the gills. The Pliocardiinae are highly specialized for sulphide-rich reducing environments, harbouring chemoautrophic bacteria in their gills. This is the first summary of the generic structure of the family Vesicomyidae that allow us to analyze the distribution of vesicomyids at the generic level. We recognize here five different distribution patterns that are related to the specific environmental demands. The general trends in the distribution patterns of the vesicomyids are an occurrence of the majority of genera in broad geographical ranges and the prevalence of near continental type of distribution.

## Introduction

Vesicomyid bivalves are a consistent component of communities that live in reducing environments such as cold seeps at continental margins [1], hydrothermal vents along mid-ocean ridges [2], or associated with organic remains [3]. Vesicomyids from sulphide-rich reducing environments are rather large, mostly several cm to more than 30 cm in size. All of these “large” vesicomyid clams studied so far live in symbiosis with sulphur-oxidizing bacteria in their gills [4]. The clams have access to substantial concentrations of hydrogen sulphide in the pore water [5], [6]. In contrast to the “large” vesicomyids, much less is known about the smaller representatives of the family, belonging to species of the type genus of the family - *Vesicomya* Dall, 1886, typified by *Vesicomya atlantica* (Smith, 1885). This genus mostly occurs in deep-sea oceanic basins and trenches. Species of this group are small, usually less than 1 cm and often only millimetric in size. Their mode of nutrition is largely unknown, although some evidence exists for the presence of bacteria in their gills [7]. Vesicomyids may thus be considered as a model taxon in order to study adaptation strategies towards a chemosynthesis-based nutrition.

The family Vesicomyidae is distributed world wide from shelf to hadal depths. This broad geographical distribution, and the wide vertical range, in combination with the high level of specialisation, makes this group a perspective study object to learn about biogeographical and ecological aspects of reducing communities. However, the confused taxonomy of the vesicomyids has so far not allowed any general picture of their global distribution to be drawn.

During the last few years substantial steps towards a robust taxonomy of the family Vesicomyidae have been made [8]–[13]. Studies based on the soft body, as well as on the shells, have revealed a high taxonomic diversity that required the separation of species into distinct genera [8], [10], [12]. In addition to the taxonomic work, there have been many new discoveries of vesicomyids in different regions of the World Ocean.

The objective of this study is to give an overview of the current taxonomy and global distribution pattern of vesicomyids at the generic level. To start with, the current taxonomic uncertainties connected to the family Vesicomyidae are summarized, followed by a short perspective of how the taxonomical problems may be solved, based on our ongoing research. In the second section, the current state of the generic composition of Recent species of the family is presented, based on published data and complemented by our investigations in progress. It also includes data on vesicomyids from newly discovered sites. The third part presents and discusses the global distribution of vesicomyid clams at the generic level based on all records that we know of, as well as the distribution pattern of the better-defined genera.

## Materials and Methods

This survey is founded on the database comprising all Recent species of vesicomyids and their occurrence. The database includes the information both from literature sources and unpublished data of the authors. Representatives of two species of the type genus *Vesicomya* were investigated by scanning electronic microscopy using standard methods of material preparation. The first species, *V. atlantica*, was collected during the cruise of R/V “Jean Charcot” (INCAL, st. OS07; 47°32′N, 09°34′W, 4249 m, 10.08.1976) and the other, as yet undescribed species was obtained during the expedition of R/V “Polarstern” (st. 080-9, 70°39.07′S 14°43.36′W, 3103 m).

Conclusions about the generic assignments of species were based on conchological and anatomical (where possible) features. The species *“Calyptogena” magnifica* Boss & Turner, 1980 and *“Calyptogena” regab* Cosel & Olu, 2009 need to be assigned to new genera, work that is currently underway. In the list of genera these taxa are designated as Genus N 1 and Genus N 2 correspondingly (Table 1). The generic assignment of another 22 described species is not yet defined. In total 87 Recent (including late Pleistocene and Holocene) species were allocated to 16 genera. In the list of genera information is given on the type species of the genus, their provisional composition of Recent species (including undescribed ones based on unpublished data of the authors), the geographical distribution and the vertical range for the genus as a whole.

**Table 1:** Summary of the vertical and geographic occurrence of genera in the family Vesicomyidae.

Genus	Vertical range, m	Western Pacific Ocean	Eastern Pacific Ocean	Western Atlantic Ocean	Central Atlantic Ocean	Eastern Atlantic Ocean	Indian Ocean	Arctic	Antarctic
*Vesicomya*	108–9530	X	X	X	X	X	X		X
*Pliocardia*	486–3159	X	X	X	X	X	X		
*Abyssogena*	2985–6400	X	X	X	X	X			
*Akebiconcha*	100–710	X							
*Archivesica*	192–2050	X	X						
*Callogonia*	900–1805			X		X	X		
*Calyptogena*	489–3136	X	X	X		X	X		
*Ectenagena*	486–502		X						
*Elenaconcha*	579–670					X			
*Isorropodon*	150–6809	X	X			X	X	X	
*Laubiericoncha*	1323–3159	X	X	X		X		X	
*Phreagena*	549–2005	X	X		X		X		X
*Wareniconcha*	754–3010	X	X			X	X		
*Waisiuconcha*	290–1445	X				X			
Genus 1	2450–3100		X						
Genus 2	3140–3170					X			
	Total number of genera in every region	11	11	6	4	11	7	2	2
	Number of endemic genera in every region	1	2	0	0	2	0	0	0
	Total number of genera in every ocean	Entire Pacific: 13		Entire Atlantic: 11			7	2	2

We do not consider genera containing exclusively fossil species (*Adulomya* Kuroda, 1931, *Hubertschenckia* Takada, 1953 and *Pleurophopsis* Van Winkle, 1919). Author's names are not indicated except for species with same species epithets. Authors and dates of species will be provided in the Checklist of described Recent and fossil Vesicomyidae (Cosel, Krylova, in preparation).

In order to describe the distribution patterns of vesicomyid genera the following types are distinguished: transoceanic (a genus that occurs on both sides of the ocean or in more than in one oceans), regional (a genus occurring in one large region of the ocean), and “point” (a genus occurring at a single locality) [14]. The distribution patterns in relation to the distance from the continents were divided into 3 types: near-continental type, oceanic type and panthalassic type, when a genus occurs in both oceanic and near-continental areas [15], [16]. For discussion on the faunistic affinities we compared the vesicomyid fauna of eight large regions of the World Ocean using a presence-absence data set (Table 1).

## Results and Discussion

### Taxonomical uncertainties related to the Vesicomyidae

#### Relationships between *Kelliella* and *Vesicomya*

**Figure 1:**
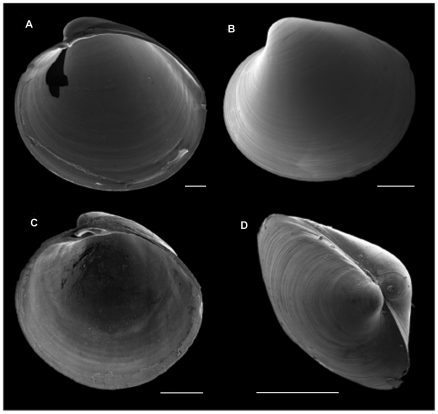
Scanning electron microscopy images of two species of *Vesicomya*. (A) Interior of right valve and (B) Exterior of left valve of *Vesicomya atlantica* (Smith, 1885) (47°32′N, 09°34′W, 4249 m). (C) Interior of right valve and (D) dorso-lateral view of *Vesicomya* sp. (70°39.07′S, 14°43.36′W, 3103 m). **Scale bar 0.5 mm.**

The family Vesicomyidae Dall & Simpson, 1891 was originally established to include only one genus: *Vesicomya* Dall, 1886, comprising small-sized bivalves characterized by heterodont dentition without lateral teeth (Figure 1). This genus has such great similarity with the genus *Kelliella* M. Sars 1870, the type genus of the family Kelliellidae P.Fischer, 1887, that the question of synonymy of these two genera has been discussed for many years. If they are synonymous, or at least belong to the same family, the family name Kelliellidae would take a priority over Vesicomyidae. In the history of studying *Kelliella* and *Vesicomya* many authors [17]–[21] have considered that they do belong to the same family, thus making the name Vesicomyidae invalid. Other authors have considered them as belonging to different families [11], [22]–[35]. A major task for taxonomists is, therefore, to resolve the relationships between *Kelliella* and *Vesicomya*, in order to determine which family name is valid. For this question it is necessary to conduct a comparative study of both genera with morphological and molecular analyses. In this paper, we keep Vesicomyidae as valid name for the family, as our ongoing investigations support the view that the two genera *Vesicomya* and *Kelliella* belong to separate families.

#### Relationships between *Vesicomya* and the “large vesicomyids”

Another problem with the taxonomy of vesicomyids is the relationships of the type genus *Vesicomya* to all other genera currently assigned to the Vesicomyidae.

**Figure 2:**
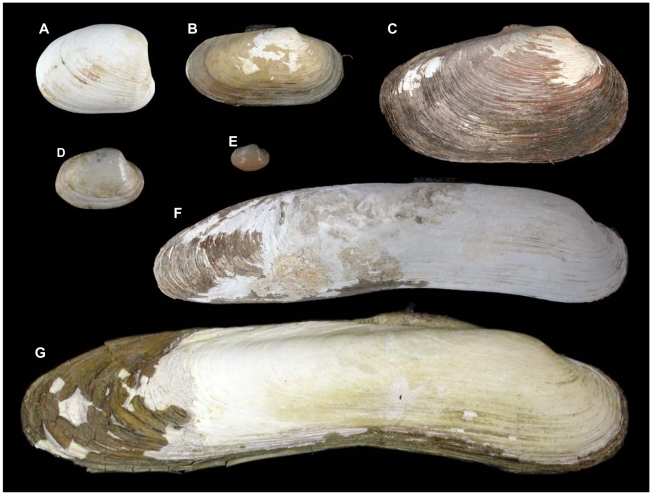
Exterior of right valves of selected pliocardiins. (A) *Pliocardia kuroshimana* (Okutani, Fujikura, Kojima, 2000) (“Shinkai 2000”, Dive 981, 24°07′N, 124°12′E, 785 m, 8 October 1997, L = 45 mm, P.P. Shirshov Institute of Oceanology). (B) *Calyptogena makranensis* Krylova, Sahling, 2006 (RV Sonne, st. 322, 24°32′N, 64°15′E, 2336 m, 28 April 1998, L = 62.8 mm,SMF 327540, holotype). (C) *Laubiericoncha chuni* (Thiele, Jaeckel, 1931) (N/O ATALANTE BIOZAIRE 2, ROV-PL 147-10, 5°47′S, 9°42′ E, 3151–3159 m, 1 December 2001, L = 93.47 mm, MNHN). (D) *Callogonia leeana* Dall, 1889 (11°40′N, 58°33′W, 1609 m, L = 33.4 mm, USNM 95423, paratype). (E) *Isorropodon bigoti* Cosel, Salas, 2001 (5°53′5S, 11°38′84′ E, 150 m, “N'Kossa” oilfield, L = 14.7 mm, P.P. Shirshov Institute of Oceanology). (F) *Abyssogena phaseoliformis* (Métivier, Okutani, Ohta, 1986) (RV *Sonne*-110, st. 28/2, 57°27.25′ N, 147°59.67′ W, 4867 m, 28 July 1996, L = 179 mm, University of Bremen). (G) *“Ectenagena” extenta* Krylova, Moskalev, 1996 (RV Keldysh, st. 2355, 36°53′3N, 122°30′5W, 3041 m, L = 229 mm, P.P. Shirshov Institute of Oceanology).

Most obviously, *Vesicomya* differs from the other vesicomyids in size (Figures 1, 2). In addition, our unpublished data show that *Vesicomya* differs by the absence of subfilamental tissue in their gills. The subfilamental tissue in all “large” vesicomyids comprises cells containing symbiotic bacteria [4]. Despite the fact that the subfilamental tissue is absent, studies using transmission microscopy showed that *Vesicomya sergeevi* Filatova, 1971, the only species of the group for which published results are available, has bacteria in its gill tissue [7]. However, it is still unclear if these bacteria are symbiotic. The nutritional basis of *Vesicomya* is, thus, largely unknown and an interesting subject for further studies.

From a taxonomic point of view, the relationship of *Vesicomya* to the “large vesicomyids” that occur at seeps and vents is significant. If *Vesicomya* is not confamilial with the other genera currently included in the family, the use of the name Vesicomyidae must be restricted to the genus *Vesicomya* only. However, our ongoing research indicates that *Vesicomya* is related to the genera of large-sized vesicomyids.

#### Generic composition

For a long time the family Vesicomyidae contained only the genus *Vesicomya*. One of the now well-known vesicomyid genera, *Calyptogena* Dall, 1891, was originally placed in the family Carditidae [35]. The genus *Pliocardia* Woodring 1925, which now is also considered to be a vesicomyid was originally assigned to the family Pliocardiidae Woodring 1925. Woodring [25] was the first to assign the genera of large-sized clams, *Calyptogena* Dall, 1891, *Phreagena* Woodring, 1938 and *Ectenagena* Woodring, 1938, to the family Vesicomyidae.

The modern shape of the family was formed to a large extent by [29], who considered as vesicomyids the following genera and subgenera: *Vesicomya*, *Callogonia* Dall, 1889, *Veneriglossa* Dall, 1886, *Waisiuconcha* Beets, 1942, *Archivesica* Dall, 1908, *Akebiconcha* Kuroda, 1943, *Calyptogena*, *Ectenagena*, *Phreagena*, *Hubertschenckia*, *Pleurophopsis*, and *Pliocardia*. Later [11] showed that *Isorropodon* Sturany, 1896 is a valid genus of Vesicomyidae, and [36] transferred to the family the genus *Adulomya*. Three more new vesicomyid genera, *Laubiericoncha*, *Wareniconcha* and *Elenaconcha* were established by [9], [10] and the description of one further genus, *Abyssogena*, is in press [12]. However, based on our unpublished results, *Veneriglossa* is not a genus of Vesicomyidae.

Estimations of the actual number of vesicomyid subgenera and genera in the recent literature vary from 1 to 13 [8], [9], [11], [28], [37]. Furthermore, molecular analysis does not support the monophyly of currently used generic names [38]–[40]. This uncertainty is apparently the consequence of ambiguous generic diagnoses, which is probably a result of morphological convergences and the common occurrence of reduction in the hinge structures. For example, characters such as strong elongation of the shell and reduction of the anterior ramus of the subumbonal cardinal tooth in the right valve have evolved independently several times in the family. The convergence in shell shape may be illustrated by *Abyssogena phaseoliformis* and *“Ectenagena” extenta* shown in Figure 2.

There has been progress, however, in establishing better-defined genera by compiling diagnostic characters including anatomical features. These include the recently revised genera *Calyptogena* and *Abyssogena* [8], [12]. Published molecular data supports the monophyly of these genera, i.e. [38]–[40] showed that species assigned to *Abyssogena*, including *phaseolifomis*, *kaikoi*, and *southwardae* (from the Atlantic Ocean) form a well-supported cluster in a phylogenetic tree based on COI sequences. Furthermore, the close relationship between species belonging to the genus *Calyptogena* is supported by COI sequences [38], [39] generally confirming the revised generic composition, with namely one exception. Due to its morphological differences, *Vesicomya lepta* has not been ascribed to the genus *Calyptogena* [8] and probably belongs to the genus *Wareniconcha*. In order to resolve the taxonomical position of *V. lepta*, additional studies of the anatomy and the variability of the shell shape are needed.

Next to the genera *Calyptogena* and *Abyssogena*, additional clusters of species exist in molecular phylogenetic trees suggesting that these are generic groups as well. However, a taxonomic revision of these clusters is needed to confirm this. For example, species such as *V. kuroshimana* and “*V*.” *crenulomarginata* are closely related to each other and are well separated from all other vesicomyid species [39]. One of the species of this natural group has been recently ascribed to the genus *Pliocardia* [13], suggesting that the other species may belong to this genus as well. A further natural cluster in phylogenetic trees is formed by species including *“V.” gigas* and *“C.” kilmeri*, termed the “*gigas/kilmeri*-complex” in [38]. Preliminary investigations by the authors revealed a morphologically complex group of several species that are currently assigned to different genera (*Archivesica, Phreagena, Akebiconcha*), which makes them a very challenging group to study.

It is worth noting that several species are well separated from the other species within the family Vesicomyidae, which is shown by unsupported branches in consensus trees based on molecular data, for example *“C.” magnifica*, *“V.” kaikoae*, or *“E.” extenta* [39], [40]. It is likely that some of these species indeed belong to new genera, for example *“C.” magnifica*, alternatively, it is possible that the relationship of already sequenced species changes when additional samples are studies.

### Taxonomy of the family Vesicomyidae

The fact that the family Vesicomyidae comprises two taxonomically distinct groups formed by *Vesicomya* in one, and the genera of “large” vesicomyids in the other, calls for a formal designation of these groups. Therefore, we propose to distinguish two subfamilies in the family Vesicomyidae: Vesicomyinae Dall & Simpson, 1891 and Pliocardiinae Woodring, 1925.

The subfamily Vesicomyinae includes only the genus *Vesicomya*, which is characterized by small body size (usually less than 10 mm), thin cardinal teeth parallel to the hinge margin, short siphons, lack of pallial sinus, non-reduced gut, two pairs of demibranchs, and an absence of subfilamental tissue in the gills. Symbiosis with chemoautotrophic bacteria has not yet been proved for *Vesicomya*, although there are some indications that bacteria are present [7].

Genera within the subfamily Pliocardiinae show different sets of characters, however their shared characters include mainly medium or large body size, well developed siphons, reduced gut, and the presence of subfilamental tissue in the gills. All representatives of the Pliocardiinae studied so far are highly specialized for sulphide-rich environments, harbouring chemoautrophic bacteria in their gills. This subfamily seems to be only found in sulphide-rich reducing habitats. The following list of genera is based on the current published taxonomy. However, the generic composition of Pliocardiinae is in flux; some genera are probably “composite” and will be split during further study, while others will be lumped. For example, the relationships between the genera *Archivesica*, *Phreagena* and *Akebiconcha* are not yet resolved. Despite the type species of these genera being clearly different, they are obviously closely related groups, and all species assigned to these genera need revision.

Family Vesicomyidae Dall & Simpson, 1891.

Subfamily Vesicomyinae Dall & Simpson, 1891.

*Vesicomya* Dall, 1886.

Type species: *Callocardia atlantica* Smith, 1885.

Distribution: throughout the Atlantic; Indian Ocean: central part and Eastern (Sunda Strait); throughout the Pacific; Antarctic: East Weddell Sea; 108–9530 m.

Provisionally assigned species: *abyssicola*, *adamsi*, *albida*, *atlantica*, *biscayensis*, *bruuni*, *concentrica*, *galatheae*, *indica* (Knudsen, 1970), *laevis*, *nitida*, *pacifica* (Smith, 1885), *profundi*, *rotunda*, *sergeevi*, *sirenkoi*, *sundaensis*, *tasmanensis*.

Subfamily Pliocardiinae Woodring, 1925.

*Pliocardia* Woodring, 1925.

Type species: *Anomalocardia bowdeniana* Dall, 1903 (Jamaica, Bowden; Late Pliocene).

Distribution: Western and Eastern Atlantic; Central Atlantic: MAR (Logatchev); Western and Eastern Indian Ocean; Western and North-Eastern Pacific; 486–3159 m

Provisionally assigned species: *atalanta*, *brevis*, *caribbea*, *crenulomarginata*, *indica* (Smith, 1904), *kuroshimana*, *solidissima* (Prashad, 1932), *ticaonica*.

*Abyssogena* Krylova, Sahling & Janssen, in press.

Type species: *Abyssogena southwardae* Krylova, Sahling & Janssen, in press.

Distribution: throughout Atlantic; Western and Eastern Pacific; 2985–6400 m.

Provisionally assigned species: *kaikoi*, *novacula*, *phaseoliformis*, *southwardae*, undescribed species from the Rucku Trench [39].

*Akebiconcha* Kuroda, 1943.

Type species: *Akebiconcha kawamurai* Kuroda, 1943.

Distribution: Western Pacific; 100–710 m.

Provisionally assigned species: type species only.

*Archivesica* Dall, 1908.

Type species: *Vesicomya gigas* Dall, 1896.

Distribution: Eastern and Western Pacific; 192–2050 m

Provisionally assigned species: *gigas*, *ochotica*, an undescribed species from off Costa-Rica.

*Callogonia* Dall, 1889.

Type species: *Callocardia (Callogonia) leeana* Dall, 1889.

Distribution: Western Atlantic: Caribbean Sea; Eastern Atlantic: off Morocco and Mauritania; Western Indian Ocean off Madagascar; 780–1805 m.

Provisionally assigned species: c*yrili*, *leeana*, *mauritanica*, an undescribed species from off Madagascar.

*Calyptogena* Dall, 1891.

Type species: *Calyptogena pacifica* Dall, 1891.

Distribution: Western Atlantic: Caribbean Sea; Eastern Atlantic: off Morocco, Gulf of Guinea; Eastern and Western Pacific; North Indian Ocean; 489–3136 m.

Provisionally assigned species: *costaricana*, *fausta*, *gallardoi*, *goffrediae*, *makranensis*, *pacifica* Dall, 1891, *rectimargo*, *starobogatovi*, *tuerkayi*, *valdiviae*, an undescribed species from the Caribbean Sea.

*Ectenagena* Woodring, 1938.

Type species: *Calyptogena elongata* Dall, 1916,

Distribution: Eastern Pacific: off California; 486–502 m.

Provisionally assigned species: type species only.

*Elenaconcha* Cosel & Olu, 2009.

Type species: *E. guiness* Cosel & Olu, 2009.

Distribution: Eastern Atlantic: off Mauritania and Gulf of Guinea; 579–670 m.

Provisionally assigned species: type species only.

*Isorropodon* Sturany, 1896.

Type species: *Isorropodon perplexum* Sturany, 1896.

Distribution: Eastern Atlantic: from off Namibia to off Mauritania; Mediterranean; North Atlantic: Norwegian Sea; North Indian Ocean; Pacific Ocean: Japan Trench, Kurile Trench, Aleutian Trench; 150–6809 m.

Provisionally assigned species: *bigoti*, *curtum*, *elongatum* (Allen, 2001), *perplexum*, *striatum*, *fossajaponica*, undescribed species from the Norwegian Sea and from the Indian Ocean.

*Laubiericoncha* Cosel & Olu, 2008.

Type species: *Laubiericoncha myriamae* Cosel & Olu, 2008.

Distribution: Western Atlantic: Caribbean Sea, Gulf of Mexico; Eastern Atlantic: Gulf of Guinea; Western Pacific: South-China Sea, Edison Seamount; Eastern Pacific: Panama Bay, Baja California, off Costa Rica; Arctic: Gakkel Ridge; 1323–3159 m.

Provisionally assigned species: *angulata*, *chuni*, *myriamae*, *nanshaensis*, *suavis*, an undescribed species from the Gakkel Ridge (Holocene).

*Phreagena* Woodring, 1938.

Type species: *Phreagena lasia* Woodring, 1938; (Los Angeles Basin, Lower Pliocene).

Distribution: Western and Eastern Pacific; Central Atlantic: MAR (Rainbow); North Indian Ocean; Antarctic: Larson Shelf; 549–2005 m.

Recent species: e*disonensis*, *kilmeri*, *nankaiensis*, *ochotensis*, *okutanii*, *soyoae*, four undescribed species from MAR (Rainbow, Pleistocene), off Costa Rica, the Larson shelf, and the Indian Ocean.

*Wareniconcha* Cosel & Olu, 2009.

Type species: *Vesicomya guineensis* Thiele & Jaeckel, 1931.

Distribution: Eastern Atlantic: Gulf of Guinea, off Cameroon and Angola margin; South -Western and Eastern Pacific; Western and North Indian Ocean; 754–3010 m.

Provisionally assigned species: c*ompressa*, *cretacea*, *guineensis*, *lepta*, *ovalis*, *winckworthi*.

*Waisiuconcha* Beets, 1942.

Type species: *Waisiuconcha alberdinae* Beets, 1942 (Indonesia, Buton Island, Late Miocene).

Distribution: Eastern Atlantic: off Mauritania; Western Pacific; 290–1445 m.

Provisionally assigned species: *haeckeli*, *helios*, *surugensis*.

Genus N 1

Distribution: Eastern Pacific: EPR, Galapagos Rift; 2450–3100 m.

Provisionally assigned species: “C.” *magnifica*.

Genus N 2

Distribution: Eastern Atlantic; 3140–4125 m.

Provisionally assigned species: “C.” *regab*.

### Distribution

#### Geographic distribution of the family

**Figure 3:**
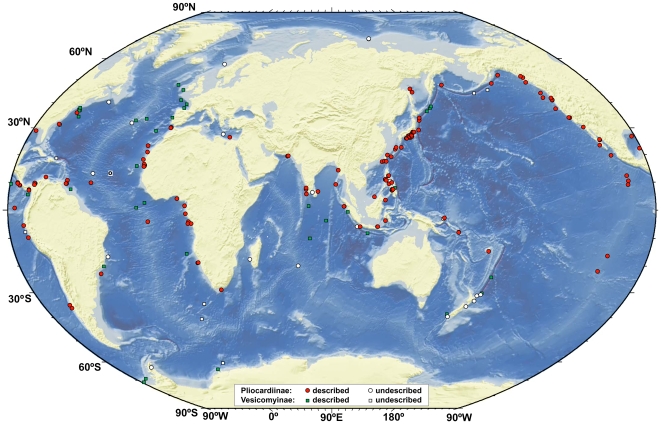
Global occurrence of the Vesicomyidae. The subfamily Pliocardiinae comprise all genera with “large vesicomyids” found at seeps and vents, the subfamily Vesicomyinae only includes the genus *Vesicomya* mainly recovered from deep trenches and ocean basins.

The map including all published and unpublished discoveries of vesicomyids illustrates the different distribution pattern of the members of subfamilies Vesicomyinae (genus *Vesicomya*) and Pliocardiinae (“large” vesicomyids) (Figure 3). Both Vesicomyinae and Pliocardiinae are distributed worldwide and have panthalassic type ranges, but vesicomyins mainly occur in trenches and in abyssal plains, whilst pliocardiins live mostly on the continental margins and along mid-ocean ridges. The fact that vesicomyins occur on abyssal plains could indicate that the subfamily probably does not depend on the presence of high concentration of hydrogen sulphide, which is in contrast to pliocardiins [5], [6]. Sulphide-rich reducing environments may occur in trenches as a result of methane seepage but there is no geochemical or geological evidence for high sulphide concentrations near the seabed in oceanic basins where some vesicomyins have been collected. We therefore conclude, that at least some vesicomyins do not depend on sulphide-rich reducing environments.

**Figure 4:**
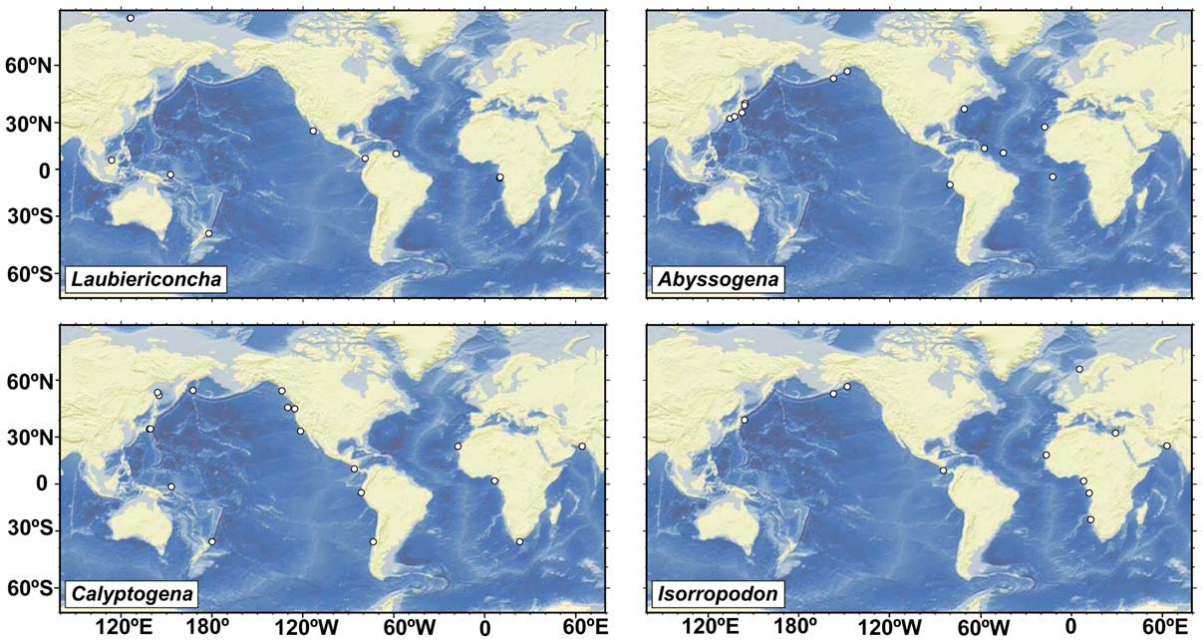
Global occurrence of the genera *Laubiericoncha*, *Abyssogena*, *Calyptogena*, and *Isorropodon*.

The known distribution of the Pliocardiinae along continental margins is remarkable, as members of the group have been recovered not only from methane seeps (see [41] for the most recent map showing the distribution of seeps with chemosynthetic communities), but also from many additional sites, e.g. along the coast of Mauritania (Western Africa), the Arctic Ocean (Intersection of Gakkel Ridge with the continental margin off Siberia), near off Madagascar and around India. The widespread occurrence of pliocardiins supports the view that reducing environments are much more common along continental margins than generally considered so far. There seem to be many geological settings that lead to methane seepage, along not only tectonically active, but also passive continental margins. Seepage along convergent margins can be very frequent as shown by the systematic discovery of 110 seeps along a 460 km long stretch of the continental slope offshore Middle America [42]. However, seepage can also be frequent along tectonically passive margins, due to various geological processes that may lead to seepage, as recently summarized for the continental margin offshore Congo, western Africa [43], [44]. Obviously, dispersion along continental margins is the predominant migration pathway for the pliocardiins. The dispersion along methane seeps near continents with the result of a worldwide distribution has been achieved by several genera in this subfamily (Figure 4).

#### Geographic distribution of the genera

The preliminary data on the generic composition of the family allows us to conduct a provisional analysis of the World distribution of vesicomyids at the generic level (Table 1). The most noticeable feature of the distribution patterns of the vesicomyid genera is the wide geographical range. Eleven genera (73%) have a transoceanic distribution, with seven genera known from three oceans and three genera from two oceans. Only five genera (27%) have regional distribution pattern. In the vesicomyids there is no genus with a “point” range distribution type.

The other notable feature of the distribution of the vesicomyids is the obvious prevalence (10 genera, or 63%) of a near-continental range pattern, which is a direct consequence of the occurrence of methane seepage at continental margins. Only five genera (31%), *Vesicomya*, *Abyssogena*, *Laubiericoncha*, *Pliocardia* and *Phreagena*, have a panthalassic distribution; Genus 1 (*“C.” magnifica*) is the only genus that occurs only in the oceanic area (at hydrothermal vents on the EPR).

The distribution pattern of vesicomyids reveals common features with the general distribution of seep fauna. As in the vesicomyids the majority of seep animal genera have wide, transoceanic distribution, with a near continental type of distribution [14]. For comparison, among obligate hydrothermal genera only 16% have transoceanic ranges [14] and 60% have very limited distribution occurring in only one hydrothermal region [45]. In addition, the hydrothermal fauna is mainly characterized by an oceanic pattern of distribution [14]. With respect to their tendency to occur near continents, vesicomyids also differ from bivalves of regular deep-sea habitats, which are mainly characterized by panthalassic distribution pattern [19]. Apart from that, there is no apparent latitudinal zonality in vesicomyids, such as a circum-tropical or high-latitude distribution pattern as documented for many regular deep-water animals.

Among all the compared regions, the highest similarity is between faunas of the western and eastern regions of the Pacific Ocean. It is likely that the only endemic genus of the Western Pacific, *Akebiconcha*, will be synonymized with the widely distributed genus *Phreagena*, and a result of this would be that the fauna of the Western Pacific will comprise only genera also found in the Eastern Pacific. On contrast to the western and eastern regions of the Pacific, the faunas of the Eastern and Western Atlantic differ considerably. Out of five genera which are known in the Eastern Atlantic and absent in the Western Atlantic, two genera are endemic of the Eastern Atlantic whereas the other three are widely distributed in the Pacific and Indian Oceans. The fauna of the Indian Ocean is poorly segregated from the faunas of the Pacific and Atlantic Oceans at the generic level and represents impoverished generic composition compared with them.

#### Vertical distribution

The vertical range of the vesicomyids as a whole is very broad extending from 108 m to 9530 m in vesicomyins, and from 100 m to 6400 m in pliocardiins (Table 1). The reports of the upper limit of pliocardiins at water depth as shallow as 100 m are remarkable, as these are further examples that vent and seep-specific species are not limited to water depth deeper than ∼200 for vents and ∼400 m for seeps, respectively. The virtual absence of common vent and seep-specific species shallower than 200 to 400 m has been noticed previously by several authors based on regional studies [46], [47] as well as literature reviews [2], [48]. The shallow finding of pliocardiins as well as reports of shallow occurrences of specialized for sulphide-rich environments modioline mussels [49] and vestimentiferan tube worms (Siboglinidae) [50] at some localities shows that the proposed ∼200 to 400 m depth limit is not a strict barrier and should be considered as a boundary describing the distribution of most but not all vent- and seep-typical species.

The vertical ranges of each genus with the family Vesicomyidae is narrow. Only two genera (*Vesicomya* and *Isorropodon*) are eurybathic, occurring from the sublittoral to the abyssal zones. Five genera are distributed throughout two zones, with two genera occurring in the sublittoral and bathyal zones, and three genera occurring in the bathyal and abyssal zones. Nine genera are restricted to a single bathymetric zone: seven genera have a bathyal distribution and two (*Abyssogena* and Genus 2) occur only in the abyssal zone (Table 1). It is interesting to notice, that either the lower or the upper limit in vertical distribution of eight genera (which amounts to 25% of all generic depths limits) is a depth of about 2500–3100 m. This depth level corresponds to the accepted limit between bathyal and abyssal zones [51].

#### Conclusion

It is possible to distinguish some general distribution patterns in the family Vesicomyidae at the genus level. The distribution pattern of the genus *Vesicomya* can be formally defined as a transoceanic, eurybathic distribution with a panthalassic type of range. This remarkably broad distribution of *Vesicomya* (Subfamily Vesicomyinae) is probably the result of a less specialized nutrition compared to those genera that exclusively occur at sulphide-rich reducing habitats. Those genera (Subfamily Pliocardiinae), which live in symbiosis with sulphide-oxidizing endosymbionts in reducing habitats may be formally divided into four groups, characterized by the following distribution patterns: (1) transoceanic mainly bathyal-abyssal distribution with near-continental range type: *Archivesica*, *Callogonia*, *Calyptogena*, *Isorropodon*, *Waisiuconcha*, *Wareniconcha,* (2) transoceanic bathyal-abyssal distribution with panthalassic range type: *Abyssogena*, *Phreagena*, *Pliocardia*, *Laubiericoncha*, (3) regional mainly upper-bathyal distribution with near-continental range type: *Akebiconcha*, *Ectenagena*, *Elenaconcha*, and (4) regional low bathyal-upper abyssal distribution with oceanic range type: *“C.” magnifica*.

We suggest that even with the further clarification of the generic structure, the general trends in the distribution patterns of vesicomyids will largely remain the same, with the occurrence of a majority of genera in broad geographical ranges and the prevalence of near continental distribution type.
